# Methods of Attaining Asepsis at Operations

**Published:** 1909-10-16

**Authors:** C. Barrett Lockwood

**Affiliations:** Surgeon to St. Bartholomew's Hospital


### Hospital Clinics.

### METHODS OF ATTAINING ASEPSIS AT OPERATIONS.

By C. BARRETT LOCKWOOD, F.R.C.S., Surgeon to St. Bartholomew's Hospital.

(A Lecture Delivered at the Hospital on Wednesday, July 21, 1909.)

Gentlemen,—I thought it would be of service to tell you what I am doing to attain asepsis—rather a difficult topic. As far as one can tell, surgeons are not agreed upon the meaning of the word " asepsis." There is a school, which has perhaps been less prominent lately, which applies the term " asepsis " to the methods which it employs: sterilised gauze, salt solution, and so forth. But I think that almost every month, certainly every year, it will have to alter its definition, because those methods do not persist. There are many things in aseptic surgery besides the ordinary means which are used. We apply the term " asepsis " to the end which we endeavour to achieve, and that is the entire absence of all bacteria, quite irrespective of their properties. I can remember the time when staphylococcus epidermidis albus was considered of no account, and perhaps harmless, in wounds. But nowadays no surgeon would call a bacterium harmless which leads to softening of blood clot and to rather violent inflammation of the tissues in its vicinity. If you accept the logical definition of asepsis, namely, the absence of all kinds of bacteria, then of course you have got a scientific test by which to ascertain whether you succeed or do not succeed. And the scientific test is the persistent use of culture media, delicate and reliable; no surgery is in proper order unless it is continually checked by the use of culture media.

I said that aseptic surgery consists in much more than a mere consideration of sterilised gauze and salt solution. Of late years attention has been more and more turned to attaining an aseptic environment for operations. It is difficult to see how surgeons can expect success with the so-called aseptic surgery if, during the use of sterilised gauze and salt solutions the atmosphere is pouring all kinds of bacteria—more particularly, in this city, dung bacteria—into the wounds. We occasionally hear of disasters which occur to surgeons who have endeavoured to carry out what they consider the acme of aseptic surgery in the atmosphere of this great city, which is shown by Dr. Gordon and others to contain, even at the height at which I am lecturing, various kinds of bacteria, some of them derived from the human person, and others from the animals which are perambulating the streets. An aseptic environment, obviously, is much more difficult to attain in a city such as this than it is in a small town in the country or half-way up a Swiss mountain.

Operating theatres are not always planned in a satisfactory manner; and if you want examples of unsatisfactory operating theatres you will not have very far to seek. Unfortunately, there is no science of ventilation. Some theatres and wards are ventilated by a system of draughts. I have often considered what is the most suitable size for an operating theatre. There is something more than the mere asepsis to be considered. The surgeon is entitled to consider his own comfort, and an atmosphere may be aseptic and yet be very uncomfortable for the human being who is breathing it. For operating with perhaps twenty people in the theatre in addition to the patient the size of a theatre ought to be from 8,000 to 10,000 cubic feet; and the atmosphere ought to be renewed at least six times in the hour. There are two ways in which the atmosphere can be renewed in an operating theatre; and should you have to alter and adapt old theatres, there is no question in my mind but that the so-called natural system is that which is most adaptable. The natural system, as you know, extracts the air, and other air is allowed to come in as best it can; by doors, ventilators, windows, or by Tobin's tubes. We have tested the air entering by the windows and by Tobin's tubes, and we find that it always contains dung bacteria; and therefore we, of necessity, have covered all these apertures with layers of gauze. How many layers of gauze should be applied I do not know. One helps; two or three are better. When I come to the question of how many layers of gauze are required to prevent the surgeon and his assistants from coughing into the wound, I should say that nothing less than eight layers is of any reliable service. But there is another system of ventilation, a system which forces air which has been purified, warmed, and moistened, into the theatres. And that system can only be applied to buildings which are specially built and adapted for it. At present I think that system is undoubtedly the best. I occasionally work in the theatre at St. Thomas's Hospital Nursing Home; not a large theatre, but one which is ventilated by the forcing-in system. That, undoubtedly, is the pleasantest theatre that I have ever had to work in. But the audience is small; in addition to the patient, there are seldom more than five or six people in the theatre. There are many objections to the forcing-in system. As you are aware, it is expensive, and requires skilled attendance. A rather complicated system which is supervised by the British working man not infrequently breaks down. In this building you are breathing air which is supplied by what is called a modified Plenum system. In the new Law Courts over the way they have a Plenum system which, about six months ago, broke down, with results which were highly unpleasant to those who had to work in those Law Courts.

One of the great sources of contamination of the atmosphere, in addition to the dust which comes in from the roads through the windows and ventilators, is undoubtedly the air passages of those in the theatre. I think you may take it as a fact that during ordinary respiration nothing is emitted from the air passages; and during ordinary conversation nothing is emitted. I say ordinary conversation, but I suppose that, taking the imperfections of human nature into account, there are few people who talk long over a wound without spluttering into it. Therefore there is no question that during the performance of an operation the surgeon should keep his mouth shut; or, if he talks, he should cover his mouth by at least eight layers of gauze. The boots are a serious source of infection. I myself have seen horse dung on the floor of the operating theatre; and, short of such gross quantities of filthy matter, there are minute particles which, of course, must be carried in upon the boots. Therefore I decline to have on the floor of the theatre any person who has not, prior to entering the theatre, covered his feet with some suitable covering. There are many coverings in vogue; gum boots or goloshes are often used. These are objectionable. I do not see how they can be sterilised, because they cannot be subjected to heat. It does not require a very powerful imagination to enable you to picture to yourselves dirty boots leaving the mud which dries on them, and this being afterwards pumped into the atmosphere by those who walk about in the boots. Perhaps it is less objectionable for the surgeon to take his boots off in another room, and then to thrust his feet into his own particular pair of gum boots, which they attempt to keep as clean as possible, not aseptic. Therefore we use coarse canvas overalls. These can be washed and sterilised.

In addition to the person of the surgeon, assistants, nurses, and so forth, being a source of infection in operating theatres, the patients and especially their clothing, are a source of infection. Some time ago, when, on account of cases of infection, having occurred, I was proceeding with this matter, Miss Stewart, the Matron, very sensibly said, "What is the use of taking all these precautions with the surgeons and others if patients are brought into the theatre in their own clothing and in the ward blankets?" Obviously that is not right. Miss Stewart then set to work and devised a proper twill sheeting, to put the patient on, and by means of which he or she could be lifted from the ambulance on to the operating table; and she made the patients come into the theatre with properly sterilised clothing on. One frequently sees patients brought into the theatre with their heads uncovered. The head of a hospital patient is not particularly aseptic; and when that has dawned on the minds of those in charge of theatres, we find patients brought down with suitable head-coverings on. Porters who bring the patients into the theatre, too, are not allowed on the floor of the theatre without suitable coverings on their persons and on their feet. But I think it would be much more satisfactory to keep porters out of the theatre entirely, and we endeavour to do that, the patient being put off the ambulance on to the operating table, which is outside, and is wheeled in by dressers or by nurses. So you can imagine I do not entirely approve of those singularly elaborate operating tables which are made fixtures in the centre of the operating theatres. I need hardly describe the preparation of patients for the operations, but I would say this: that in addition to a bath and properly sterilised clothing, and in addition to the necessary shaving, I never, outside the hospital, put on a preparatory dressing. The preparatory dressing which is put on over night is singularly inefficacious; it makes the patient very uncomfortable, and I think, by making the skin more sodden, it makes it more difficult to disinfect. But we know, from a long sequence of tests, that by shaving and thoroughly washing the skin with soap and water, and by soaking it in the 1 in 500 biniodide and spirit, alternating with 1 in 500 of a watery solution, we get a very high proportion of aseptic skin, that is to say, a skin which yields no result when it is put into broth. I may say the same of the hands of the surgeon and of his assistants. If the nails are cut short, if the hands are properly washed with hot water and soap, and if for some three to five minutes they are treated alternately with 1 in 500 of biniodide and spirit and 1 in 500 of biniodide and water, you can get so much mercury into the skin that the person may have mercurial poisoning. We have found mercury in the skin of the hands twenty-seven hours after it had been prepared. I observe that many surgeons—and myself amongst others—are addicted to the use of rubber gloves. I dislike rubber gloves very much indeed. I have found myself taking them off when engaged in a difficult operation which involved the separation of adhesions in the iliac fossa or in the pelvis. I also find it very difficult to sew with rubber gloves on. And gloves frequently get torn. So you see the surgeon must be an expert in hand disinfection; and that disinfection is not by any means an easy task.

I will not trouble you with some of the trifling details of aseptic surgery which are performed by nurses and others, but I will proceed now to say a word upon the use of ligatures. To my mind there is no ligature which can surpass the ordinary twisted silk; but, as a rule, too large is used. Even for thick pedicles I have found that the smaller sizes, 1, 2, or 3, give a more efficient hold than the very thick. I suppose silk is seldom put into the human body without a certain amount of bacterial contamination; and the bacteria have a better breeding place in thick than in very thin silk. But whether that is so or not, better results have been obtained since the more minute sizes of silk were more frequently employed. But I sometimes see silk put into the wrong kinds of wounds. I have seen silk put into wounds which are obviously septic. If there is the slightest chance of septic contamination in a wound, there is no question that it is wiser to put in fine catgut, not chromicised, but the sort which is calculated to last five to seven days; and I not infrequently see silk put into wounds which are obviously tuberculous. Yesterday I was occupied in removing glands which were tuberculous from the neck of a boy. Some of them were burst, and some caseous material went into the tissues. But no silk was put into the wound. Some tissues, like the peritoneum, or the aponeurosis of the external oblique, bear the presence of silk extraordinarily well. Silk is not at all well borne by muscular fibres. When after appendicectomy silk is extruded, it is almost always the piece of silk which is put on the fibres of the internal oblique and transversalis. For muscular fibres I use chromicised catgut, which is calculated to last 20 to 30 days. And many times when I have been putting that chromicised catgut on the muscular fibres some bystander has said, "You have omitted to pull your loop tight." That omission is intentional, for the simple reason that those muscles are going to move; and experience has shown me that if you put ligatures of any kind on muscular fibres which move, you will very likely see them again. Since I have adopted the plan of putting chromic gut on the muscular fibres and not pulling the loop tight, I have had a singular absence of suppuration. I need hardly say anything about fishing-gut. We use it in wounds which are likely to have been contaminated at the time of operation. For instance, in cases of gastro-enterostomy, after splitting the rectus abdominis and pulling it apart, and it has to be brought together again, this should be used. The ideal method of uniting layer after layer takes rather a long time in a patient who may have been exhausted by a long course of vomiting and malnutrition. And moreover, during the course of gastro-enterostomy it is so very common for some gastric or duodenal contents to be smeared in the field of the operation. Therefore, under such circumstances, I use fishing-gut, which is easily disinfected by heat.

I now come to the question of the use of wire or other foreign body in aseptic surgery. Here I think you should make up your mind as regards the principle upon which you are about to act. I observe that some surgeons use wire and splints and screws and nails, and such-like appliances, with, as it appears to me, an entirely mechanical idea in their mind. That is, supposing the patella is to be fastened together, the wire is put in under the notion that it will act mechanically and prevent those fragments coming apart and will withstand the strain of the quadriceps extensor femoris. I do not believe it is possible by mechanical means to obtain such security that the contraction of one of the most powerful muscles in the body can be resisted. And I have observed that surgeons who use wire with the idea that it is going to resist this enormous strain have had curious things happen to their patients. You have heard of the patient who, at the end of six weeks has pulled his patella apart, and not only so, has pulled his wound apart, and his knee-joint has been found exposed. I have operated upon patients who, a short time after having had wire or other substance introduced, have been allowed to resume their ordinary avocations and have speedily pulled the fragments apart again. The idea that I have in my mind is, first, to expose the fracture; and then to prevent anything being left between the broken surfaces. After that, I proceed merely to put in enough wire to bring the clean bony surfaces into perfect bony apposition, and keep them there until repair has taken place. The limb is kept absolutely still for a month in hospital. After that, it is put in plaster, and the patient usually goes to a convalescent home for another month. He is not allowed to use it at his work, if that work is of a laborious kind, for another month. So that three months are consumed in the effort to get bony union. The other day, one of our patients who had gone through that routine got into a tramcar, and something gave way in his knee. It was obvious when he came back that he had pulled his patella apart. I think the mistake we made in that instance was this: before that man was allowed to go along the streets and jump on a tramcar, his knee ought to have been photographed again, to see whether or not there was bony union. So I use wire and such-like appliances, not with any mechanical notion in my mind, but to keep bony surfaces in apposition until they have undergone their proper process of repair.

I need hardly say anything about swabs or sponges. You may have observed that sponges are used less and less: there is nothing so good for clearing a wound as an ordinary marine sponge; but there is also nothing more dangerous unless you can either disinfect it yourself or can trust the people who do disinfect it. And the disinfection of a marine sponge is a laborious process. And, therefore, we have discarded sponges more and more. In using sponges it is necessary to use some kind of lotion. Operations are begun with the dry swabs, but before the end of the operation there will be dried blood in the vicinity of the wound, which cannot be removed with dry swabs, and so some lotion must be used. Sterilised water would be a very good lotion; so also would normal salt solution; but in this city, where we are so doubtful about the asepticity of our environment, we still use dilute antiseptics. The one I am in the habit of using is biniodide of mercury, but doubtless there are others equally efficacious. A good deal of wild talk has been emitted as to the harm which chemicals do to the human tissues. I cannot help thinking that it is founded on the use of 1 in 1,000 perchloride of mercury, or 1 in 20 carbolic lotion. Those, of course, applied to the tissues, produce a layer of necrosis; but the dilute chemicals which we use—1 in 4,000 biniodide, sometimes 1 in 6,000 or 1 in 10,000—do not do harm; and I have looked at microscopic sections of the tissues before and at the end of an operation, and have been unable to tell the difference. I advise everyone to continue to use dilute chemicals until they are absolutely certain they are operating with scientific accuracy and in a scientifically aseptic environment.

Drainage we use more than formerly. I often ask why a fluid called blood has ever been put into the human body, because this fluid, when it escapes from the vessels, clots. If it clots in the tissues, it inflames them. Everyone here is aware that an effusion of blood in the peritoneal cavity inflames the peritoneum so much that the attack of peritonitis is often thought to be suppurative. I am prepared to admit that an effusion of blood into the peritoneal cavity frequently becomes infected and softened, and although it has not the physical characters of pus, certainly has the potentialities of pus. But why should the body contain a fluid which, when extravasated into the tissues, can be so exceedingly harmful? I think few realise fully the effects of blood and blood clot on the human tissues, upon the serous and synovial membranes. Hence it is that one takes care to clamp vessels when they are divided, and remove the blood from the wound as quickly as possible. All sorts of extraordinary things are invented for draining wounds; but I observe that people have, after all, come back to the rubber drainage tube. Our drainage tubes are put in to let out the blood, and they are usually taken out at the end of 48 hours. I might say that in dressing the wound the dressing is so arranged that the drainage tube can be extracted without removing more than a part of the dressing. For instance, after the complete operation for removal of the breast, with removal of the pectorals and the axillary lymphatic glands, the drainage tube was put rather low down in the mid-axillary line, and an additional dressing is placed there, to be taken away at the time of the extraction of the tube, so that the rest of the dressing is never touched. It is objectionable to expose wounds to the atmosphere of this city or of the wards of the hospital, so we do all we can to avoid this exposure, and when the dressing is done we endeavour to get it done as quickly as we can. Our wounds are generally dressed about the eighth or ninth day. I observe that people are too fussy about the removal of fishing-gut sutures. If we use such sutures, we endeavour to leave them in until they have begun to cut, because, if taken out too soon, they do not assist much in the consolidation of the scar; and aseptic fishing-gut is not a very harmful substance—and even if it is I do not think it matters much if it leads to a better consolidation of the scar. But when the wound is dressed we do all we can to have a proper scientific test made at the time. The suture is put into a broth tube; or, if any blood or moisture is seen, that is at once tested. And in the wards under my charge nobody would dream of saying to me, "This wound has suppurated," unless he was prepared at the next moment to tell us what the infection was. Pus is but the sign of an infection, and, of course, now it is absolutely essential to know what the infection is; because a knowledge of the kind of infection may be followed by a course of treatment by vaccines or by therapeutic sera.

I have no fixed ideas about my methods of wound treatment. I am prepared to abandon or alter any of them to-morrow; but before I do so I shall require proper scientific proof that the value of my former treatment is less than that of the one suggested.

